# 6-Isoprenylindole-3-carboxylic Acid with an Anti-Melanogenic Activity from a Marine-Derived *Streptomyces* sp. APA-053

**DOI:** 10.3390/md23120448

**Published:** 2025-11-21

**Authors:** Minjeong Kim, Eun-Young Lee, Ga-Eun Shin, Jungwook Chin, Hongchan An, Sang-Jip Nam, Kyung-Min Lim

**Affiliations:** 1 College of Pharmacy, Ewha Womans University, Seoul 03760, Republic of Korea; tyndall@ewha.ac.kr (M.K.); younglee0124@naver.com (E.-Y.L.); gaeun5136@ewhain.net (G.-E.S.); 2 Cureverse, Inc., H2 Building, KIST, Seoul 02792, Republic of Korea; jwchin@cureverse.co.kr; 3 New Drug Development Center, Daegu-Gyeongbuk Medical Innovation Foundation, Daegu 41061, Republic of Korea; hongchanan@cha.ac.kr; 4 College of Pharmacy and Institute of Pharmaceutical Sciences, CHA University, 120 Haeryong-ro, Pocheon 11160, Republic of Korea; 5 Department of Chemistry and Nanoscience, Ewha Womans University, Seoul 03760, Republic of Korea; 6 Graduate Program in Innovative Biomaterials Convergence, Ewha Womans University, Seoul 03760, Republic of Korea

**Keywords:** marine-derived actinomycetes, *Streptomyces* sp., anti-melanogenic activity, melanocyte, antioxidant activity, skin-brightening, 3D models

## Abstract

In this study, we report the isolation of the known compound 6-isoprenylindole-3-carboxylic acid (SJ196), a prenylated indole derivative, from a marine *Streptomyces* sp., APA053, and its potent anti-melanogenic activity. SJ196 showed ABTS and DPPH radical scavenging activities and cellular antioxidant activities, significantly suppressing cytoplasmic and mitochondrial reactive oxygen species (ROS) in B16F10 murine melanoma cells. Furthermore, SJ196 reduced both intracellular and extracellular melanin content without cytotoxicity. These effects coincided with suppression of intracellular signal transduction for melanogenesis, significantly reducing phosphorylation of ERK, JNK, and p38 MAPK, and attenuating the expression of MITF and melanogenic enzymes (TYR, TRP-1, and TRP-2). Importantly, in a three-dimensional human skin model (MelanoDerm™), SJ196 exhibited a skin-lightening effect, as evidenced by dose-dependent increases in skin brightness and histological confirmation. Collectively, we demonstrated that SJ196 is a potent anti-melanogenic marine compound that acts through antioxidant activity and MAPK-MITF pathway suppression, suggesting its therapeutic potential for the treatment of age-related hyperpigmentation disorders.

## 1. Introduction

Marine actinomycetes, especially *Streptomyces* species, represent a prolific reservoir of structurally diverse and biologically active metabolites [1]. Their ecological adaptation to marine environments is reflected in distinctive biosynthetic pathways that yield specialized metabolites with antimicrobial, anticancer, immunomodulatory, and enzyme-inhibitory properties [2,3]. The metabolic repertoire of marine *Streptomyces* is further distinguished by structural features such as halogenation and prenylated indole frameworks, which are rarely observed in terrestrial counterparts [4]. Several studies have reported indole-containing natural products from marine-derived *Streptomyces*, highlighting their potential as a rich source of novel chemotypes with therapeutic relevance [4,5].

Recent advances in marine natural products research have underscored the genus *Streptomyces* as a particularly promising source of anti-melanogenic agents [6,7]. Certain *Streptomyces* species are known to produce structurally unique secondary metabolites that interfere with melanin biosynthesis [7,8], a process of central importance in both hyperpigmentation disorders and cosmetic applications frequently occurring in the aged population. A notable example is deoxyvasicinone, a tricyclic quinazoline alkaloid isolated from *Streptomyces* sp. CNQ-617. This compound has been shown to markedly suppress melanin accumulation [9]. Such findings highlight the potential of marine-derived *Streptomyces* as a valuable source of bioactive agents capable of modulating melanogenesis.

Skin pigmentation is regulated by the balance between melanin synthesis and its distribution, and disruption of this equilibrium can lead to hyperpigmentation disorders, resulting in both cosmetic and psychological burdens [10]. At the biochemical level, melanin biosynthesis depends on the catalytic activity of tyrosinase (TYR) and its related enzymes, tyrosinase-related protein-1 (TRP-1) and TRP-2, whose transcription is controlled by the master transcriptional regulator of melanogenesis, microphthalmia-associated transcription factor (MITF). Increasing evidence indicates that, in addition to enzymatic regulation, oxidative stress—particularly reactive oxygen species (ROS)—acts as a critical upstream inducer of melanogenic signaling pathways [11,12].

During the process of melanogenesis, tyrosinase-mediated oxidation reactions generate ROS as by-products, thereby exposing melanocytes to a significant level of oxidative stress [13]. Under physiological conditions, endogenous antioxidant systems, including glutathione, thioredoxin, and catalase, mitigate ROS accumulation to prevent aberrant signaling [14]. However, when oxidative stress overwhelms cellular defense mechanisms, ROS activate mitogen-activated protein kinase (MAPK) pathways—such as ERK, JNK, and p38—as well as other kinases, which enhance MITF transcription, stability, and activity. Consequently, the expression of TYR, TRP-1, and TRP-2 is amplified [15,16]. This feedforward interaction suggests that inhibiting tyrosinase alone, without alleviating oxidative stress, may be insufficient to effectively suppress hyperpigmentation.

Given this mechanistic association, antioxidants capable of scavenging ROS can serve as dual-action depigmenting agents, not only neutralizing ROS but also downregulating subsequent signaling cascades. Many natural compounds, such as flavonoids and polyphenols, act within this paradigm as radical scavengers that modulate intracellular signaling and enzymatic activity [17,18]. Nevertheless, limitations such as low potency, chemical instability, poor skin permeability, and restricted target specificity often constrain their clinical applicability. To overcome these challenges, synthetic or semi-synthetic derivatives inspired by natural scaffolds have been developed through strategic structural modifications aimed at improving bioavailability, efficacy, and multimodal activity [17,19,20].

In this study, we report the isolation of 6-isoprenylindole-3-carboxylic acid (**1**), a prenylated indole derivative, from a marine-derived *Streptomyces* sp. APA-053. While SJ196 has been found in terrestrial microorganisms, we report for the first time its isolation from a marine-derived *Streptomyces* sp. SJ196 is characterized by an aromatic or heterocyclic core, a conjugated alkene bridge, and a terminal carboxylic acid moiety. This structure exhibits molecular features favorable for radical stabilization via delocalization, as well as potential metal coordination interactions such as hydrogen bonding or copper chelation within tyrosinase’s active site. We further investigated the potential of SJ196 as a dual-function skin-lightening agent capable of scavenging ROS while simultaneously suppressing melanogenesis through modulation of signaling and enzymatic pathways, using both B16F10 melanoma cells and a reconstructed pigmented human epidermis model (Melanoderm™).

## 2. Results and Discussion

### 2.1. Isolation and Structure Elucidation of 6-Isoprenylindole-3-carboxylic Acid

6-Isoprenylindole-3-carboxylic acid (Compound **1**) was isolated from the fermentation of the marine-derived strain APA-053 obtained from moss collected on Jeju Island in July 2020, South Korea. Analysis of the 16S rRNA gene sequence (accession number PQ651499) revealed 99.9% similarity to *Streptomyces malachitospinu* (NR041423.1), leading to its identification as a member of the genus *Streptomyces* sp. Detailed extraction methods were described in Materials and Methods.

Compound **1**, a white amorphous powder, exhibited a protonated molecular ion peak at *m*/*z* 230.28 [M + H]^+^ in the low-resolution electrospray ionization tandem mass spectrometry (LR-ESI-MS). The ^1^H NMR spectrum for compound **1** showed two methyl singlet protons [H-4′ (*δ*_H_ 1.75, s, 3H) and H-5′ (*δ*_H_ 1.76, s, 3H)], one methylene proton [H-1′ (*δ*_H_ 3.44, d, *J* = 7.5 Hz, 2H)], two methine protons [H-2 (*δ*_H_ 7.87, d, *J* = 1.5 Hz, 1H), and H-2′ (*δ*_H_ 5.36, t, *J* = 7.3 Hz, 1H)], and three aromatic protons [H-4 (*δ*_H_ 7.94, d, *J* = 8.1 Hz, 1H), H-5 (*δ*_H_ 7.01, dd, *J* = 8.1, 1.5 Hz, 1H), and H-7 (*δ*_H_ 7.22, br s, 1H)] (Figure S1). The ^13^C NMR spectroscopic data displayed 14 carbon signals, comprising one carboxylic acid carbon C-1′′ (*δ*_C_ 169.3), five quaternary carbons [C-3 (*δ*_C_ 108.6), C-6 (*δ*_C_ 137.7), C-8 (*δ*_C_ 125.6), C-9 (*δ*_C_ 138.6), and C-3′ (*δ*_C_ 132.9)], three aromatic and two methine carbons [C-2 (*δ*_C_ 133.0), C-4 (*δ*_C_ 121.8), C-5 (*δ*_C_ 125.1), C-7 (*δ*_C_ 111.9), and C-2′ (*δ*_C_ 123.5)], one methylene carbon [C-1′ (*δ*_C_ 35.3)], and two methyl carbons [C-4′ (*δ*_C_ 17.9), and C-5′ (*δ*_C_ 25.9)] (Figure S2).

**Figure 1:**
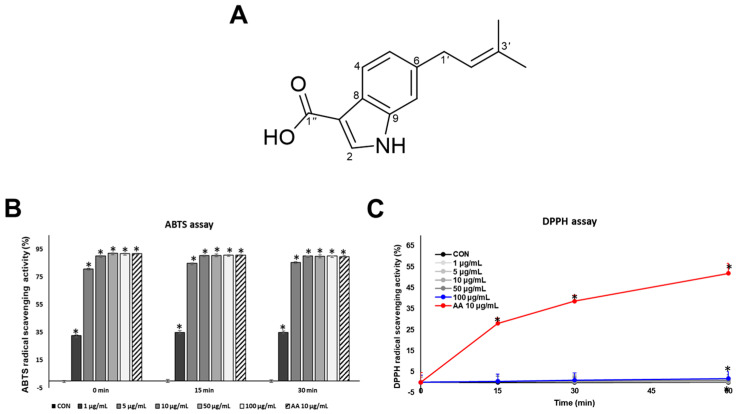
Chemical structure of SJ196 and its antioxidant activity evaluated by ABTS and DPPH assays: (**A**) Chemical structure of SJ196. Antioxidant activity was determined using (**B**) the ABTS assay and (**C**) the DPPH assay. Radical scavenging activity was measured spectrophotometrically at 517 nm. Ascorbic acid (AA, 10 μg/mL) was used as a positive control. Data are presented as mean ± SD (*n* = 4; * for *p* < 0.05 vs. control).

Based on the comparison of NMR data with those reported in the literature, compound **1** was identified as 6-isoprenylindole-3-carboxylic acid (Figure 1) [21]. According to the previous studies, this compound exhibited weak antibacterial activity against *Bacillus subtilis*, *Staphylococcus aureus*, *Sarcina lutea*, and *Pseudomonas* sp. [21]. It was also reported to possess fungistatic properties toward the crop-pathogenic fungi *Gaeumannomyces graminis* var. *tritici*, *Rhizoctonia cerealis*, *Helminthosporium sativum*, and *Phytophthora capisici* [22].

### 2.2. Antioxidant Effects of SJ196 in a Cell-Free System

The antioxidant activity of SJ196 (Figure 1A) was evaluated using the ABTS and DPPH radical scavenging assays, with ascorbic acid (AA, 10 μg/mL) serving as the positive control. The antioxidant properties of SJ196 can be attributed to its structural features. The indole ring provides an electron-rich π-system that stabilizes unpaired electrons, while the conjugated prenyl group enhances radical resonance stabilization. The terminal carboxylic acid contributes further through hydrogen bonding and potential metal ion chelation [18]. By virtue of these characteristics, SJ196 is endowed with the potential to scavenge free radicals efficiently and consequently regulate redox balance. ABTS is soluble in both aqueous and organic media, and it is suitable for assessing both hydrophilic and lipophilic antioxidants and is generally faster and more sensitive than DPPH. In contrast, DPPH is poorly soluble in water but readily dissolves in organic solvents, making it more appropriate for evaluating lipophilic antioxidants. By performing both assays, the antioxidant capacity of SJ196 was comprehensively and accurately assessed. In the ABTS assay, SJ196 showed a significant, concentration-dependent radical scavenging activity (RSA%) compared with the control (Figure 1B). In the DPPH assay, SJ196 showed weaker activity (Figure 1C), reflecting that SJ196 may be only active in an aqueous environment.

The observed differences between the two assays likely stem from the combined effects of solubility, solvent polarity, and reaction mechanisms intrinsic to each radical system [23]. Structurally, 6-isoprenylindole-3-carboxylic acid contains a hydrophilic carboxylic acid group and a hydrophobic isoprenyl substituent, conferring a moderately lipophilic yet amphiphilic nature. Computational estimation of the partition coefficient (LogP ≈ 2.3–2.6; ChemAxon/ACD prediction) supports this intermediate polarity. Given that the ABTS assay is conducted in an aqueous or mixed aqueous–ethanolic medium, whereas the DPPH assay operates in a strictly organic phase, the stronger activity of SJ196 in the ABTS system suggests enhanced stabilization of its radical intermediates under polar conditions. This finding implies that the N–H group of the indole ring, a plausible donor site for proton or electron transfer, more efficiently participates in radical scavenging within a protic environment [24]. Such solvent-dependent differences in reactivity are consistent with the influence of solvent polarity on hydrogen-atom transfer (HAT) and single-electron transfer (SET) mechanisms [23].

### 2.3. Inhibitory Effects of SJ196 on Melanin Production and Tyrosinase Activity

**Figure 2:**
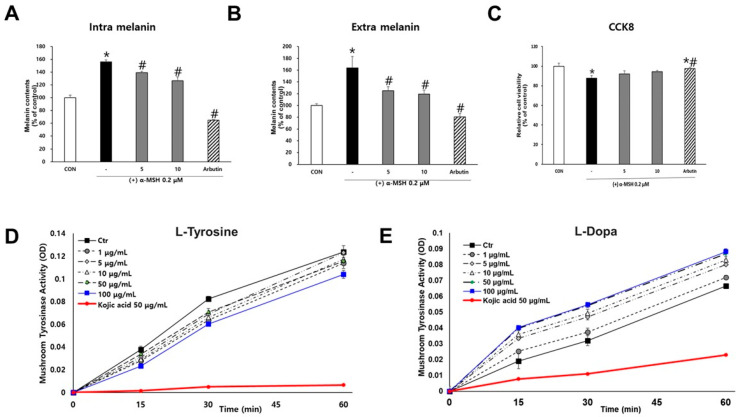
Antimelanogenic activity of SJ196 in cell-based and cell-free systems. B16F10 cells stimulated with 0.2 μM α-MSH were treated with SJ196 for 48 h: (**A**,**B**) Melanin content was quantified using a melanin assay, and (**C**) cell viability was assessed by CCK-8 assay. Tyrosinase activity was measured in a cell-free system using mushroom tyrosinase with (**D**) L-tyrosine and (**E**) L-DOPA as substrates. Kojic acid (KA, 50 μg/mL) was used as a positive control. Data are presented as mean ± SD of OD values (*n* = 4; * for *p* < 0.05 vs. control; # for *p* < 0.05 vs. α-MSH group).

To assess the anti-melanogenic activity of SJ196, melanin content was measured in α-MSH–stimulated B16F10 cells treated with SJ196. Arbutin (50 μg/mL) was used as the positive control. Following preliminary cytotoxicity screening across various concentrations, 5 μg/mL and 10 μg/mL were selected for subsequent experiments, as these concentrations maintained cell viability above 80% (Figure 2C). Analysis of intracellular and extracellular melanin revealed that co-treatment with α-MSH and SJ196 significantly and dose-dependently reduced melanin production compared with α-MSH treatment alone (Figure 2A,B). To further investigate the inhibitory effect of SJ196 on tyrosinase—the rate-limiting enzyme in melanin biosynthesis—mushroom tyrosinase assays were performed. Kojic acid (KA, 50 μg/mL) served as the positive control. In the L-tyrosine-based assay, SJ196 exhibited a weak inhibitory activity when compared with that of kojic acid (Figure 2D). In the L-DOPA-based assay, kojic acid markedly inhibited tyrosinase activity, whereas SJ196 did not show significant inhibition relative to the control (Figure 2E), suggesting that SJ196 has only limited inhibitory activity against tyrosinase activity.

Although SJ196 exhibited only weak inhibition of mushroom tyrosinase in the cell-free assays, intracellular modulation of tyrosinase activity cannot be excluded. SJ196 markedly reduced intracellular reactive oxygen species and attenuated MAPK–MITF signaling, pathways that are well established to regulate tyrosinase transcription, protein stability, and post-translational activation [25,26]. In addition, the indole ring and carboxylic acid functionality of SJ196 provide structural elements capable of hydrogen bonding and potential coordination with active-site copper ions, a mechanism implicated in the inactivation of tyrosinase by several natural inhibitors [27]. Therefore, SJ196 likely exerts anti-melanogenic effects through a combined mechanism: predominant downregulation of tyrosinase expression via antioxidant-mediated suppression of ROS/MAPK–MITF signaling, together with possible partial interference with catalytic activity via redox modulation or weak metal chelation.

Reactive oxygen species (ROS) are well characterized as upstream modulators of mitogen-activated protein kinase (MAPK) signaling, acting both by oxidative modification of signaling proteins and by reversible inactivation of phosphatases that normally terminate MAPK activation [28]. Hydrogen peroxide and derived reactive species can oxidize the catalytic cysteine residues in protein tyrosine phosphatases (PTPs) and MAPK phosphatases (MKPs), converting the active thiol to sulfenic/sulfinic forms and thereby transiently diminishing phosphatase activity and prolonging MAPK phosphorylation [29,30]. In addition, ROS can directly modulate upstream MAP3Ks and MAP2Ks or alter thiol-dependent regulatory proteins such as thioredoxin/ASK1 complexes, further promoting ERK, JNK, and p38 activation under oxidative stress [28,31]. In our system, α-MSH increased intracellular and mitochondrial ROS in B16F10 cells concomitant with elevated p-ERK, p-JNK, and p-p38, while co-treatment with SJ196 lowered ROS levels and restored MAPK phosphorylation toward baseline, supporting the model that antioxidant activity of SJ196 contributes to normalization of redox-sensitive MAPK signaling and downstream MITF suppression.

### 2.4. Effect of SJ196 on Dendrite Formation in B16F10 Cells

**Figure 3:**
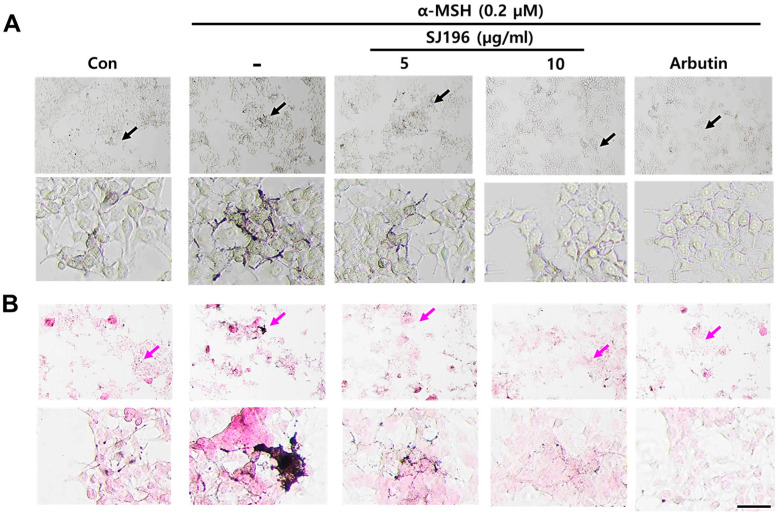
Effect of SJ196 on dendrite formation in B16F10 cells: (**A**) Morphological changes in B16F10 cells were observed following 48 h of SJ196 treatment (black arrows indicate cells with enlarged morphology). (**B**) Morphological changes were visualized by light microscopy (100×) after Fontana–Masson staining (pink arrows indicate melanocytes exhibiting extended dendrites). Scale bar = 50 μm.

During melanogenesis, melanocytes extend dendrites to transfer melanosomes to keratinocytes, and multiple intracellular signaling pathways can influence these morphological changes [32]. Thus, alterations in B16F10 cell morphology can serve as indicators of pigmentation and melanocyte activation. To examine morphological changes, B16F10 cells were treated for 48 h and observed under a microscope. Cells treated with α-MSH alone exhibited dark pigmentation and extensive dendritic elongation, whereas co-treatment with α-MSH and SJ196 led to a concentration-dependent retraction of dendrites (Figure 3A). These morphological changes were consistent with the results of Fontana–Masson (FM) staining, which showed a marked, dose-dependent reduction in melanin pigmentation in cells treated with α-MSH and SJ196 (Figure 3B).

### 2.5. Effects of SJ196 on α-MSH-Induced ROS Generation in B16F10 Cells

**Figure 4:**
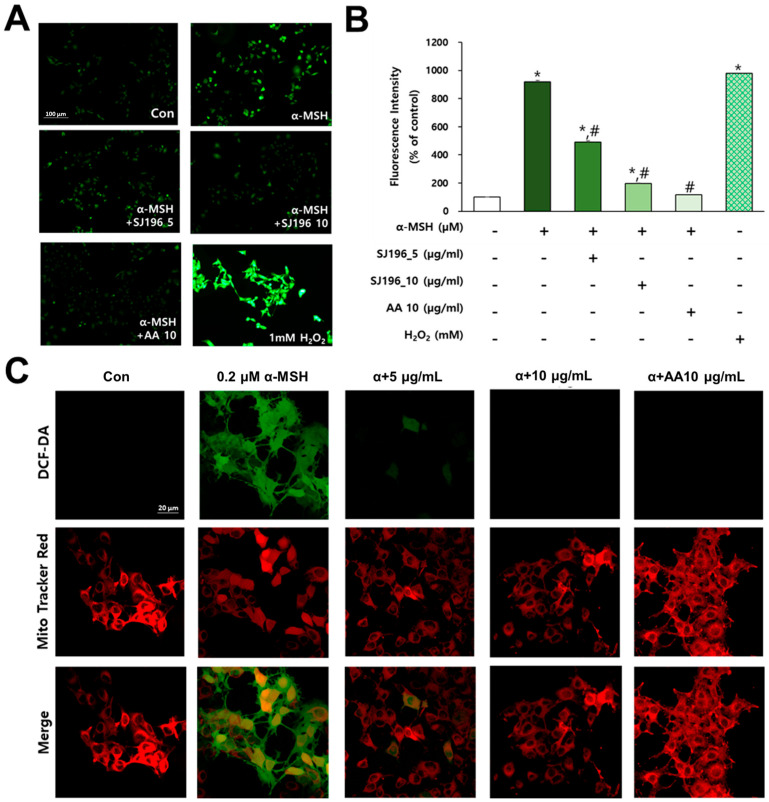
Effect of SJ196 on α-MSH-induced ROS production in B16F10 cells. Cells treated with α-MSH alone served as a negative control, and cells treated with AA (10 μg/mL) served as a positive control. B16F10 cells were treated with 0.2 μM α-MSH and SJ196 for 48 h: (**A**) ROS levels were visualized using DCF-DA fluorescence staining (100×). Bar = 100 μm. (**B**) Fluorescence intensity was quantified using ImageJ software (https://imagej.net/ij/, accessed on 1 August 2025). (**C**) Double staining with DCF-DA (green) and MitoTracker (red) was performed, and representative images were obtained by confocal microscopy (400×). Bar = 20 μm. Data are presented as mean ± SE (*n* = 3; * for *p* < 0.05 vs. control; # for *p* < 0.05 vs. α-MSH group).

To evaluate the antioxidant activity of SJ196 in melanocytes, cells were treated with α-MSH alone or co-treated with α-MSH and SJ196, followed by staining with DCF-DA and MitoTracker Red. Ascorbic acid (AA, 10 μg/mL) was used as the positive control. Fluorescence intensity was quantified using ImageJ software (https://imagej.net/ij/, accessed on 1 August 2025) (Figure 4B). DCF-DA staining revealed that SJ196 effectively and dose-dependently inhibited (Figure 7ii) intracellular ROS generation in α-MSH-stimulated cells (Figure 4A and Figure 7i). To further localize ROS suppression by SJ196, dual staining with DCF-DA and MitoTracker Red was performed and imaged via confocal microscopy. The merged fluorescence images demonstrated that SJ196 reduced total intracellular ROS levels in α-MSH-stimulated cells (Figure 4C). While both SJ196 and AA possess antioxidant potential, they act through distinct mechanisms. AA primarily serves as a direct electron donor, neutralizing reactive oxygen species and regenerating oxidized antioxidants [33]. In contrast, SJ196 stabilizes free radicals via its conjugated indole–prenyl structure and modulates intracellular oxidative signaling pathways, including MAPK–MITF, leading to reduced ROS accumulation and melanogenic gene expression. Collectively, these findings suggest that SJ196 mitigates oxidative stress by decreasing intracellular ROS accumulation in melanocytes stimulated with α-MSH.

### 2.6. SJ196 Suppresses Gene and Protein Expression Related to Melanogenesis

**Figure 5:**
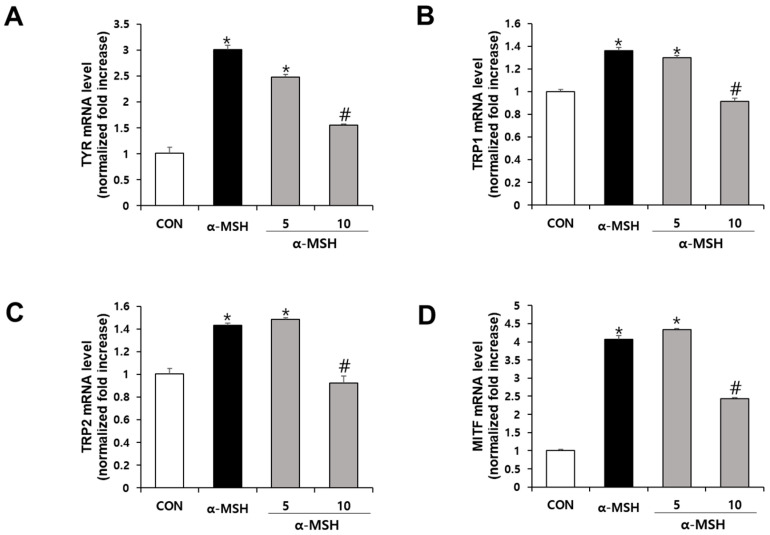
Effect of SJ196 on the mRNA expression of melanogenesis-related genes in B16F10 cells. B16F10 cells stimulated with 0.2 μM α-MSH were treated with SJ196 (5 and 10 μg/mL) for 72 h. Cells treated with α-MSH alone served as a negative control. mRNA expression levels of TYR (**A**), TRP-1 (**B**), TRP-2 (**C**), and MITF (**D**) were quantified by real-time PCR. β-actin was used as an internal reference. Data were normalized to β-actin expression. Values are presented as mean ± SE (*n* = 3; * for *p* < 0.05 vs. control; # for *p* < 0.05 vs. α-MSH group).

**Figure 6:**
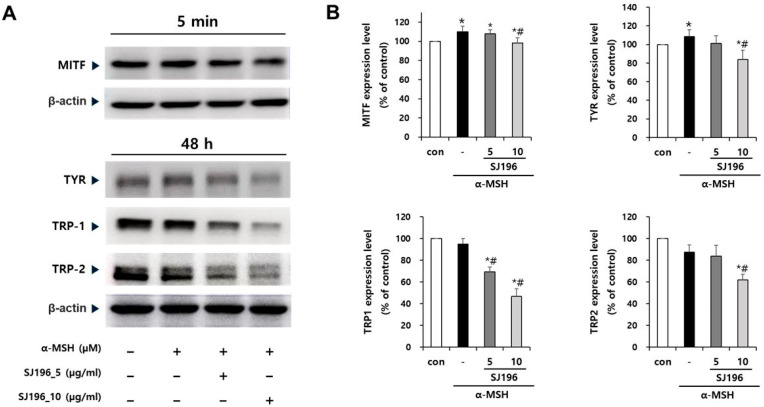
Effect of SJ196 on protein expression of melanogenesis-related targets in B16F10 cells. B16F10 cells stimulated with 0.2 μM α-MSH were treated with SJ196 (5 and 10 μg/mL) for 48 h (TYR, TRP-1, TRP-2) or 5 min (MITF). Cells treated with α-MSH alone served as a negative control: (**A**) Protein levels of TYR, TRP-1, TRP-2, and MITF were assessed by Western blotting. (**B**) Quantification of band intensities was performed using ImageJ software. β-actin was used as a loading control. Data were normalized to β-actin expression and are presented as mean ± SE (*n* = 3; * for *p* < 0.05 vs. control; # for *p* < 0.05 vs. α-MSH group).

**Figure 7:**
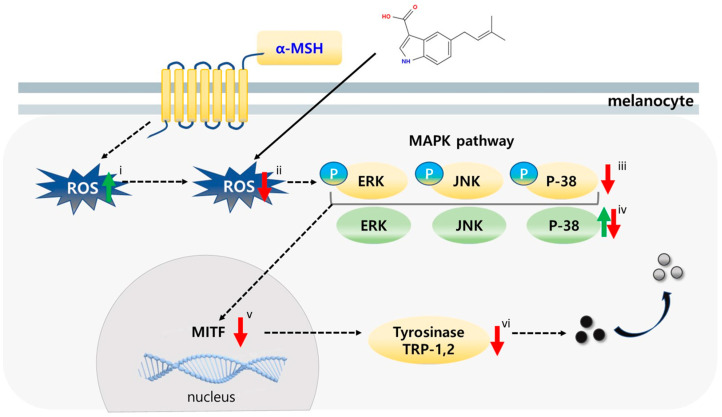
Proposed mechanism of action of SJ196 in the regulation of melanogenesis.

The effects of SJ196 on the expression of melanogenesis-related genes and proteins, namely tyrosinase (TYR), tyrosinase-related protein 1 (TRP-1), tyrosinase-related protein 2 (TRP-2), and microphthalmia-associated transcription factor (MITF), were evaluated by real-time PCR and Western blot analyses. As quantified in Figure 5, treatment with α-MSH alone significantly increased the mRNA expression of TYR (Figure 5A), TRP-1 (Figure 5B), TRP-2 (Figure 5C), and MITF (Figure 5D). In contrast, co-treatment with α-MSH and SJ196 significantly and dose-dependently downregulated TYR, TRP-1, TRP-2, and MITF mRNA expression (Figure 5 and Figure 7v,vi). Similarly, the Western blot results revealed that TYR, TRP-1, and TRP-2 protein levels were markedly decreased in a dose-dependent manner in SJ196-treated groups, while MITF protein expression was slightly but noticeably reduced at the higher concentration (Figure 6 and Figure 7v,vi). α-MSH is well known to promote melanogenesis through activation of the cAMP–PKA–CREB–MITF axis, which transcriptionally induces TYR, TRP-1, and TRP-2 [34]. Nevertheless, the final abundance of these enzymes is influenced by multiple post-transcriptional and post-translational mechanisms, including mRNA stability, translational efficiency, and proteasomal degradation that can modulate protein turnover independently of transcription [35,36]. These regulatory processes explain why α-MSH significantly enhanced the mRNA expression of TRP-1 and TRP-2 (Figure 5) without producing a proportionate rise in their protein levels (Figure 6). These results suggest that SJ196 effectively suppresses the expression of key enzymes involved in melanogenesis.

### 2.7. SJ196 Attenuates Phosphorylation of MAPK Signaling Molecules

**Figure 8:**
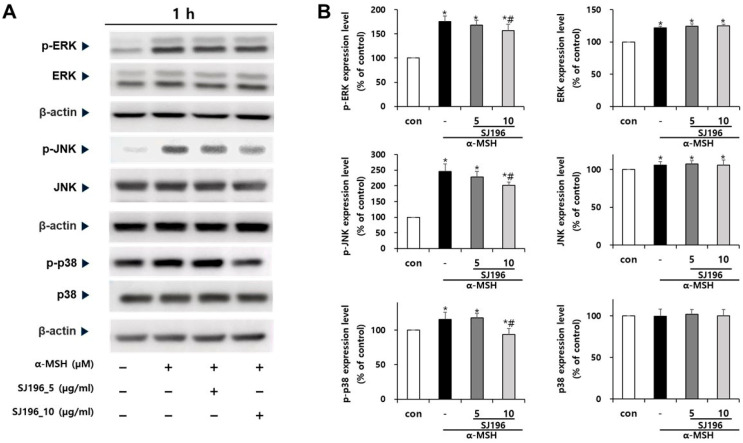
Effect of SJ196 on MAPK (ERK, JNK, and p38) phosphorylation in α-MSH-stimulated B16F10 cells. Cells stimulated with 0.2 μM α-MSH were treated with SJ196 (5 and 10 μg/mL) for 1 h. α-MSH alone served as a negative control: (**A**) Protein expression levels of total and phosphorylated ERK, JNK, and p38 were analyzed by Western blotting. (**B**) Quantitative analysis was performed using ImageJ software. β-actin was used as a loading control. Data are normalized to β-actin expression and are presented as mean ± SE (*n* = 3; * for *p* < 0.05 vs. control; # for *p* < 0.05 vs. α-MSH group).

To investigate the regulatory effects of SJ196 on mitogen-activated protein kinase (MAPK) signaling—one of the key pathways involved in melanocyte stimulation—the expression and phosphorylation of ERK, JNK, and p38 were analyzed. The expression of total ERK and JNK remained elevated in both α-MSH-treated cells and those co-treated with SJ196 compared to the control, whereas p38 expression exhibited variable patterns of increase or decrease (Figure 7iv and Figure 8A,B). In contrast, the phosphorylated forms of these kinases (p-ERK, p-JNK, and p-p38) were markedly increased after 1 h of α-MSH stimulation but were dose-dependently suppressed when co-treated with SJ196 (Figure 7iii and Figure 8A,B). These findings indicate that SJ196 modulates melanogenesis by significantly attenuating the phosphorylation of key components of the MAPK signaling cascade, including ERK, JNK, and p38.

### 2.8. Skin-Lightening Effect of SJ196 in a Human Epidermal Model (MelanoDerm™)

**Figure 9:**
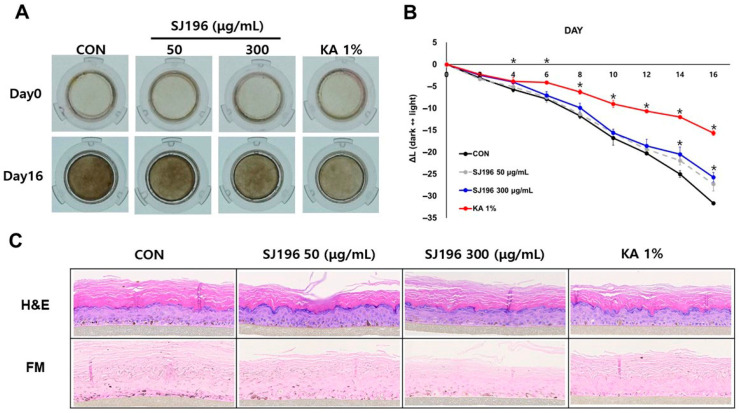
Effect of SJ196 on Melanoderm™, a 3D human pigmented epidermal model. Melanoderm™ tissues were treated with SJ196 (50 and 300 μg/mL) or KA (1%) every two days for 16 days. KA (1%) served as a positive control: (**A**) Macroscopic tissue color was evaluated on days 0 and 16. (**B**) Brightness (ΔL values) was measured every two days from day 0 to day 16. (**C**) On day 16, tissues were subjected to H&E and Fontana–Masson staining. Data are presented as mean ± SE (*n* = 3; * for *p* < 0.05 vs. control).

The human pigmented epidermal model MelanoDerm™ serves as a valuable system for studying skin pigmentation [37]. To evaluate the whitening efficacy of SJ196, the compound was topically applied to the MelanoDerm™ model at concentrations of 50 μg/mL and 300 μg/mL every other day. Changes in pigmentation were observed up to day 16 compared with day 0 (Figure 9A). SJ196 was dissolved in PBS containing 0.5% DMSO as the vehicle to ensure uniform solubility and minimize solvent effects. A volume of 30 μL of each test solution was topically applied per tissue every other day for 16 days, corresponding to the duration of the experimental cycle. The exposure period was continuous between applications, with tissue maintenance medium replaced at each dosing interval. The L* value, representing skin brightness, increased more prominently at the higher concentration (300 μg/mL) than at the lower concentration (50 μg/mL), indicating a reduction in melanin content (Figure 9B). On day 16, tissue sections were fixed and analyzed by hematoxylin and eosin (H&E) and Fontana–Masson (FM) staining. Histological evaluation revealed that melanin granules were smaller and less densely distributed in all SJ196-treated tissues compared with controls (Figure 9C). These findings demonstrate that SJ196 may be used as a skin-lightening cosmetic ingredient.

## 3. Materials and Methods

### 3.1. General Experimental Procedures

NMR spectra were obtained using Me_4_Si as an internal reference on Agilent 400-MR DD2 instruments operating at 400 and 100 MHz (Agilent Technologies, Santa Clara, CA, USA) using solvent methanol-*d*_4_ (Cambridge Isotope Laboratories (CIL), Inc., Tewksbury, MA, USA, NFEC-2021-08-272465) equipped at Ewha Drug Development Research Core Center. UV spectra were recorded on Scinco UVS2100 (SCINCO, Seoul, Republic of Korea), and VCD spectra were measured with a BioTools dualPEM Chiral *IR* spectrophotometer (BioTools, Inc., Jupiter, FL, USA). Low-resolution LC-MS were acquired using an Agilent Technology 1260 quadrupole (Agilent Technologies, Santa Clara, CA, USA) and Waters Alliance Micromass ZQ LC-MS system (Waters Corp, Milford, MA, USA) equipped with a reversed-phase column (Phenomenex Luna C18 (2) 100 Å, 100 mm × 4.6 mm, 5 µm) (Phenomenex, Torrance, CA, USA) operated at a flow rate of 1.0 mL/min at the National Research Facilities and Equipment Center (NanoBioEnergy Materials Center) at Ewha Womans University.

### 3.2. Source and Identification of Strain APA-053

The marine-derived strain APA-053 was obtained from moss collected on Jeju Island in July 2020, South Korea. Analysis of the 16S rRNA gene sequence (accession number PQ651499) revealed 99.9% similarity to *Streptomyces malachitospinu* (NR041423.1), leading to its identification as a member of the genus *Streptomyces* sp.

### 3.3. Fermentation, Extraction, and Purification

The strain APA-053 was incubated in 640 Ultra Yield flasks (2.7 L capacity), each filled with 1 L of medium composed of soluble starch (10 g/L), yeast (2 g/L), peptone (4 g/L), and artificial sea salt (34.75 g/L) in distilled water. Cultivation was carried out at 27 °C with agitation at 120 rpm for 7 days. The resulting 36 L of culture broth was extracted with EtOAc, and the combined extracts were evaporated under reduced pressure to furnish 13.22 g of crude organic extract.

The crude organic extract (13.22 g) was subjected to a C18 resin column, eluted stepwise with 1000 mL portions of H_2_O/CH_3_OH mixtures (20%, 40%, 50%, 70%, 80%, 100%, and 100% MeOH, *v*/*v*) to generate eight fractions (F1–F8). Fraction 3 (F3, 492 mg), fraction 4 (F4, 592 mg), and fraction 5 (F5, 1.17 g) underwent further purification by reversed-phase HPLC (Phenomenex Luna C-18 (2), 250 × 10 mm, 2.0 mL/min, 5 μm, 100 Å, UV = 280 nm) under isocratic elution with 48% aqueous acetonitrile containing 0.1% TFA, affording 6-isoprenylindole-3-carboxylic acid (**1**, 70.1 mg, *t*_R_ = 28.0 min).

6-isoprenylindole-3-carboxylic acid (**1**): White amorphous powder; ^1^H and ^13^C NMR data (400 and 100 MHz, methanol-*d*_4_); LR-ESI-MS *m*/*z* 230.28 [M+H]^+^.

### 3.4. ABTS and DPPH Radical Scavenging Assays

A 7 mM ABTS (2,2′-azino-bis(3-ethylbenzothiazoline-6-sulfonic acid)) solution was prepared in phosphate-buffered saline (PBS), and a 2.45 mM potassium persulfate solution was also prepared in PBS. These two solutions were mixed in a 1:1 ratio in a 50 mL conical tube, immediately protected from light, and allowed to react at room temperature for 16 h. The following day, the mixture was diluted with PBS to obtain a 4% working solution. This solution was dispensed into a 96-well plate and further adjusted with PBS until the absorbance reached 0.700 (±0.002) at 734 nm, which was used as the standard for all subsequent experiments. To evaluate antioxidant activity, 200 μL of each test sample was mixed with 800 μL of the absorbance-adjusted ABTS working solution and incubated at room temperature for 0, 15, and 30 min. The final concentrations of SJ196 were 1, 5, 10, 50, and 100 μg/mL. The assay was performed following previously described methods [38,39]. After incubation, absorbance was measured at 734 nm using a microplate spectrophotometer, with PBS (200 μL) as the solvent control.

For the DPPH radical scavenging assay, a 100 μM DPPH (2,2-diphenyl-1-picrylhydrazyl) solution was prepared in ethanol under light-protected conditions. The final concentrations of SJ196 were 1, 5, 10, 50, and 100 μg/mL, while ascorbic acid (AA, 10 μg/mL) served as the positive control. The assay was conducted according to a previously established method [40]. Briefly, 247.5 μL of DPPH solution was dispensed into each well of a 96-well plate, followed by the addition of 2.5 μL of the test sample. The plate was then protected from light and incubated at room temperature for 0, 15, 30, and 60 min. After incubation, the decrease in absorbance at 517 nm—corresponding to the reduction of the purple DPPH radical to its yellow-colored form—was measured using a BioTek Epoch microplate reader (Epoch, Biotech Instruments, Agilent, Chicago, IL, USA) [41]. Ethanol (250 μL) was used as the solvent control.

### 3.5. Cell Culture

B16F10 melanoma cells were obtained from the American Type Culture Collection (ATCC, Manassas, VA, USA). The cells were cultured in Dulbecco’s Modified Eagle’s Medium (DMEM; ATCC) supplemented with 10% fetal bovine serum (FBS; ATCC) and 1% penicillin–streptomycin (Hyclone, South Logan, UT, USA). Cultures were maintained in a humidified incubator at 37 °C with 5% CO_2_. When the cell confluence reached approximately 80%, adherent cells were detached using 0.05% trypsin–EDTA solution (Gibco, Thermo Fisher Scientific, Waltham, MA, USA) for subculture or experimental use.

### 3.6. Cell Viability Assay

Cell viability was determined using the Cell Counting Kit-8 (CCK-8; Dojindo Laboratories, Kumamoto, Japan) according to the manufacturer’s instructions. Cells were seeded in 48-well plates and treated with SJ196 at concentrations of 5 and 10 μg/mL. Cells treated with 0.2 μM α-melanocyte-stimulating hormone (α-MSH) served as the negative control, while those treated with 50 μg/mL arbutin served as the positive control. After 48 h of incubation, the culture medium was removed, and 130 μL of diluted CCK-8 solution was added to each well. The plates were protected from light and incubated for 3 h at 37 °C. Following incubation, 100 μL of each reaction mixture was transferred to a 96-well plate, and absorbance was measured at 450 nm using a BioTek Epoch microplate reader (Epoch, Biotech Instruments, Agilent, Chicago, IL, USA).

### 3.7. Melanin Content Assay

B16F10 cells were seeded in 48-well plates at a density of 2.0 × 10^4^ cells per well and allowed to adhere for 24 h. Test compounds were diluted in phenol red-free medium and applied for 48 h. Cells treated only with 0.2 μM α-MSH served as the negative control, whereas those treated with 50 μg/mL arbutin served as the positive control. SJ196 was administered at final concentrations of 5 and 10 μg/mL in medium containing 0.2 μM α-MSH. To determine extracellular melanin content, 150 μL of the culture supernatant was transferred to a 96-well plate, and absorbance was measured at 405 nm using a microplate reader. For intracellular melanin quantification, the remaining medium was removed, and 200 μL of 1 M NaOH solution was added to each well to lyse the cells. The plates were then covered to protect from light and incubated in a dry oven at 60 °C for 1 h. Subsequently, 100 μL of each lysate was transferred to a 96-well plate, and absorbance was measured at 405 nm using a microplate spectrophotometer, Infinite M200 Pro microplate reader (NFEC-2021-08-272460), Tecan Group Ltd., Mannedorf, Switzerland, equipped at Ewha Drug Development Research Core Center. A 40 mg/mL NaOH solution (100 μL) was used as a blank control, and the melanin content was expressed as a percentage relative to the untreated control group.

### 3.8. Mushroom Tyrosinase Activity Assay

The tyrosinase inhibitory activity of the test compound was evaluated using L-tyrosine and L-DOPA as substrates in a 96-well plate format. A 0.1 M potassium phosphate (PP) buffer was prepared by diluting 1 M PP stock solution with distilled water. Substrate solutions were freshly prepared under light-protected conditions to final concentrations of 0.3 mg/mL L-tyrosine and 2 mg/mL L-DOPA in 0.1 M PP buffer. Mushroom tyrosinase (500 U) was diluted with 0.1 M PP buffer to achieve optimal enzymatic activity for each assay: 250 U (enzyme–buffer = 1:1) for the L-tyrosine assay and 50 U (enzyme–buffer = 1:9) for the L-DOPA assay. Kojic acid dissolved in DMSO at a final concentration of 50 ppm served as a positive control. SJ196 was also dissolved in DMSO under identical conditions and tested at final concentrations of 1, 5, 10, 50, and 100 μg/mL. For each reaction, 180 μL of substrate solution (L-tyrosine or L-DOPA) was dispensed into each well, followed by 2 μL of the test compound solution. For the blank control, 20 μL of 0.1 M PP buffer was added instead of the enzyme solution, whereas for the experimental wells, 20 μL of tyrosinase solution (final activities: 250 U for L-tyrosine and 50 U for L-DOPA) was added to initiate the reaction. Dopachrome formation was monitored immediately using a microplate reader at 475 nm at 0, 15, 30, 45, and 60 min. During incubation, the plate was protected from light and maintained at 37 °C with orbital shaking at 350 rpm.

### 3.9. Fontana–Masson (FM) Staining

Melanin was visualized as dark black deposits using a commercial Fontana–Masson staining kit (ScyTek Laboratories, Logan, UT, USA), which enables histological visualization of dendritic melanocyte structures. Staining was performed after 48 h of treatment with the test compounds. Cells were fixed with a solution containing 5% glacial acetic acid and 95% ethanol, followed by exposure to a preheated ammoniacal silver solution. The samples were incubated for 30 min until a brownish coloration developed. After rinsing twice with distilled water, cells were briefly immersed in 0.2% gold chloride solution for 30 s, followed by additional washes and a 60 s immersion in 5% sodium thiosulfate solution. The cells were then rinsed with water for 2 min and washed twice with distilled water. Nuclear counterstaining was performed with Nuclear Fast Red solution for 5 min, followed by two rinses in distilled water. To promote dehydration, cells were passed through three sequential changes of fresh absolute ethanol.

### 3.10. Intracellular Reactive Oxygen Species (ROS) Measurement

Intracellular ROS levels were quantified using 2′,7′-dichlorodihydrofluorescein diacetate (DCF-DA; Invitrogen, Eugene, OR, USA) fluorescence staining. DCF-DA was first dissolved in anhydrous DMSO at a concentration of 500 µM and then diluted with PBS to a final concentration of 5 µM. B16F10 cells were seeded in 35 mm culture dishes at a density of 1 × 10^5^ cells per 2 mL of medium. After pre-treatment with 0.2 µM α-melanocyte-stimulating hormone (α-MSH), cells were exposed to SJ196 for 48 h. Following incubation, cells were stained with DCF-DA solution for 10 min to assess intracellular ROS accumulation. Ascorbic acid (AA, 10 μg/mL) served as a negative control, while cells exposed to 1 mM hydrogen peroxide (H_2_O_2_) for 1 h before staining served as a positive control for ROS induction. Fluorescence images were captured using a Nikon Ts2R-FL microscope (Nikon Corporation, Tokyo, Japan) equipped with NIS-Elements BR 4.6 software.

### 3.11. Mitochondrial Superoxide Detection

Changes in intracellular ROS levels and mitochondrial distribution were visualized using confocal laser scanning microscopy. Mitochondrial superoxide generation was assessed through dual staining with MitoTracker Red (Invitrogen, Eugene, OR, USA) and 2′,7′-dichlorodihydrofluorescein diacetate (DCF-DA; Invitrogen, Eugene, OR, USA). Both dyes were first dissolved in anhydrous DMSO at 1 mM and subsequently diluted in HBSS to final concentrations of 10 μM for DCF-DA and 250 nM for MitoTracker Red. B16F10 cells were seeded at a density of 1 × 10^4^ cells in 400 μL per well on a 4-well culture plate, treated with 0.2 μM α-MSH, and subsequently exposed to SJ196 for 48 h. As a positive control, 10 μg/mL of ascorbic acid (AA) was used. After incubation, cells were stained for 10 min and gently washed once with HBSS. The stained cells were mounted using Fluoromount g and imaged with a confocal microscope (Zeiss LSM 880 with Airyscan, Carl Zeiss, Oberkochen, Germany, NFEC-2016-05-209580) at the Ewha Womans University Fluorescence Core Imaging Center.

### 3.12. RT-PCR Analysis

To evaluate mRNA expression levels of Tyr, TRP1, TRP2, and MITF, B16F10 cells were seeded at a density of 2 × 10^5^ cells in 2 mL per well on a 6-well plate and treated with SJ196 for 72 h. After washing each well with phosphate-buffered saline (PBS), cells were lysed using TRIzol reagent (Invitrogen, Waltham, MA, USA). Chloroform was added, and samples were centrifuged at 12,000 rpm for 10 min at 4 °C to collect the aqueous phase. RNA was precipitated with isopropanol, mixed by inversion, and centrifuged at 12,000 rpm for 15 min at 4 °C. The supernatant was carefully removed, and the pellet was washed with 70% ethanol, followed by another centrifugation at 12,000 rpm for 5 min at 4 °C. The ethanol was then discarded, and the RNA pellet was dissolved in RNase-free diethyl pyrocarbonate (DEPC)-treated water. RNA concentration and purity were determined using a NanoDrop spectrophotometer (NanoDrop ND-1000, Thermo Fisher Scientific, Wilmington, DE, USA). Complementary DNA (cDNA) was synthesized from 375 ng of total RNA using the PrimeScript™ RT Master Mix kit (TaKaRa, Shiga, Japan). Relative mRNA expression levels were quantified via real-time PCR on a StepOnePlus™ Real-Time PCR System (Applied Biosystems, Foster City, CA, USA) using Power SYBR Green PCR Master Mix (Applied Biosystems, Foster City, CA, USA). The forward and reverse primer sequences for each gene are listed below.

β-actin: 5′-AGG GAA ATC GTG CGT GAC AT-3′ and 5′-GGA AAA GAG CCT CAG GGC AT-3′

Tyrosinase: 5′-GGG CCC AAA TTG TAC AGA GA-3′ and 5′-ATG GGT GTT GAC CCA TTG TT-3′

TRP-1: 5′-GTT CAA TGG CCA GGT CAG GA-3′ and 5′-CAG ACA AGA AGC AAC CCC GA-3′

TRP-2: 5′-TTA TAT CCT TCG AAA CCA GGA-3′ and 5′-GGG AAT GGA TAT TCC GTC TTA-3′

MITF: 5′-AGC GTG TAT TTT CCC CAC AG-3′ and 5′-TAG CTC CTT AAT GCG GTC GT-3′

Expression levels of target genes were normalized to the housekeeping gene β-actin. Cycling parameters included incubation at 60 °C for 60 s; afterward, an initial denaturation step was carried out at 95 °C for 10 min. It was then denatured at 95 °C for 15 s and annealed/extended 40 times at 60 °C for 60 s.

### 3.13. Western Blot Analysis

SDS-PAGE and Western blot analyses were conducted to assess the translational activation of target proteins. To evaluate protein expression following SJ196 treatment, B16F10 cells were seeded at a density of 2 × 10^5^ cells per 2 mL (48 h) or 4 × 10^5^ cells per 2 mL (5 min or 1 h) in 6-well plates and treated with SJ196 for 5 min, 1 h, or 48 h. After treatment, lysis buffer containing RIPA buffer, one protease inhibitor cocktail tablet, and one phosphatase inhibitor cocktail tablet was added to each well. The cells were scraped and centrifuged at 12,000 rpm for 10 min at 4 °C. Supernatants were collected, and protein concentrations were quantified using a BCA protein assay. Samples were prepared by mixing 14 ng of protein with lysis buffer, 4× loading dye, and 10× reducing agent, followed by denaturation at 95 °C. Proteins were separated via 10% sodium dodecyl sulfate–polyacrylamide gel electrophoresis (SDS-PAGE) and transferred onto membranes using the Trans-Blot Turbo Transfer System and Transfer Pack (Bio-Rad, Hercules, CA, USA). Membranes were blocked with 5% bovine serum albumin (BSA) for 1.5 h at room temperature, incubated with primary antibodies diluted in 5% BSA overnight at 4 °C, and washed three times with 1× Tris-buffered saline containing 0.1% Tween 20 (TBST). Subsequently, membranes were incubated with HRP-conjugated secondary antibodies diluted in 5% BSA for 1 h at room temperature. After additional washes, protein bands were detected using the ECL Western Blotting Detection Reagent (Cytiva, Marlborough, MA, USA) and visualized using an Amersham Imager 600 (GE Healthcare Life Sciences, Stevenage, UK). BLUEstain™ Protein Ladder (GoldBio, St. Louis, MO, USA; 11–245 kDa) was used as a molecular weight marker. β-Actin (ab8226; Abcam, Cambridge, UK) served as the internal control. The following antibodies were used: anti-tyrosinase (ab170905), anti-TRP-1 (ab235447), anti-TRP-2 (ab221144), anti-MITF (ab20663) from Abcam; and anti-p-ERK (4370S), ERK (4695S), p-JNK (9251S), JNK (9252S), p-p38 (4511S), and p38 (9212S) from Cell Signaling Technology (Danvers, MA, USA).

### 3.14. MelanoDerm™: Human Pigmented Epidermal 3D Model

The MelanoDerm™ model (MatTek, Ashland, MA, USA), consisting of normal human melanocytes and keratinocytes, was obtained from MatTek Corporation (Ashland, MA, USA). Upon arrival, the skin equivalents were stabilized for 18 h in maintenance medium (EPI-100-NMM-113) at 37 °C in a humidified incubator with 5% CO_2_ prior to the experiment. Following preincubation, tissues were treated every other day for 16 days with SJ196 or 1% kojic acid (KA), which served as the positive control. Tissue pigmentation was evaluated by photographing the samples before each treatment, and changes in brightness (ΔL values) were quantified. After 16 days, tissues were fixed in 10% neutral buffered formalin and analyzed using hematoxylin–eosin (H&E) and Fontana–Masson staining.

### 3.15. Histological Staining

For hematoxylin–Eosin staining, paraffin sections were made at 4 μm in thickness using a microtome (RM2235, Leica, Wetzlar, Germany). Formalin-fixed paraffin-embedded (FFPE) slides were deparaffinized in xylene (X0120, SAMCHUN, Seoul, Republic of Korea) 3 times and rehydrated with successive (3 × 5 min) washes in 100%, 96%, 80%, and 70% ethanol. They were then stained with hematoxylin Gill Ⅲ (H314, BIOSCIENCES, St. Louis, MO, USA), rinsed with running tap water for 1 min, rinsed with 1% hydrochloric acid, rinsed with 0.2% ammonium solution (392685, Sigma, Burlington, MA, USA) for 3 min, rinsed with tap water for 5 min, stained with 0.25% eosin (Sigma, 318906) for 4 min 20 s, and rinsed again with tap water. The slides were then dehydrated 95% for 1 min 3 times and 100% for 1 min 3 times using ethanol successively, followed by xylene (2 × 5 min), and mounted with coverslips by an automatic coversplipping machine (ClearVue Coverplipper, Epredia, Kalamazoo, MI, USA). All tissues were analyzed slide scanner (MoticEasyScan One, Motic, Waltham, MA, USA).

For Fontana–Masson Stain (For Argentaffin Cells and Melanin), paraffin sections were prepared at 4 μm thickness using a microtome (Leica, RM2235). Formalin-fixed paraffin-embedded (FFPE) slides were deparaffinized in xylene (SAMCHUN, X0120) three times, followed by rehydration through a graded ethanol series (100%, 96%, 80%, and 70%; 3 × 5 min each) and rinsing in distilled water. Staining was performed using a Fontana–Masson staining kit (FMS-IFU, ScyTek Laboratories, Logan, UT, USA) according to the manufacturer’s instructions. Briefly, slides were incubated in pre-warmed Ammoniacal Silver Solution (58–60 °C) for 30–60 min until tissue sections developed a yellowish-brown color (melanin: ~30 min; argentaffin granules: ~50–60 min). Slides were rinsed in distilled water (3 changes), treated with gold chloride solution (0.2%) for 30 s, rinsed again in distilled water (3 changes), and incubated in sodium thiosulfate solution (5%) for 1–2 min. After rinsing in running tap water (2 min) and distilled water (2 changes), slides were counterstained with nuclear fast red solution for 5 min, rinsed again, dehydrated rapidly in absolute alcohol (3 changes), cleared, and mounted with synthetic resin.

### 3.16. Statistical Analysis

All data are expressed as mean ± standard deviation (SD) or standard error (SE). Statistical significance was determined using Student’s *t*-test, and differences were considered significant at *p* < 0.05. Significant differences are indicated with asterisks or hash symbols (*, # *p* < 0.05).

## 4. Conclusions

In this study, we systematically investigated the physiological activities of a newly identified extract, designated as SJ196, across multiple melanogenesis models to elucidate its antioxidant properties, its effects on reactive oxygen species (ROS) generation, MAPK phosphorylation, MITF-dependent transcription, and its ability to modulate pigmentation outcomes.

SJ196 significantly enhanced ABTS radical scavenging activity in a dose-dependent manner [24]. In contrast, its radical-scavenging capacity in the DPPH assay was comparatively weak, a difference largely attributable to the physicochemical character of SJ196. DPPH is typically employed in organic or semi-organic media and is poorly soluble in water [41], whereas ABTS•^+^ is soluble in both aqueous and organic media, enabling the assessment of both hydrophilic and lipophilic antioxidants [23]. Numerous studies have demonstrated that ABTS often yields higher or more sensitive antioxidant values than DPPH for identical samples, due to its greater accessibility and faster reaction kinetics [42]. Since SJ196 is not strongly lipophilic, it disperses poorly in DPPH assay media, resulting in weaker apparent activity. However, its higher compatibility with aqueous systems allows more effective interaction and clearer antioxidant responses in the ABTS assay. These findings emphasize the importance of employing multiple assay systems to comprehensively evaluate antioxidant properties.

In α-MSH-stimulated melanocytes, SJ196 markedly suppressed intracellular ROS, as determined by DCF-DA fluorescence [43] and MitoTracker Red staining. α-MSH treatment alone significantly increased green fluorescence intensity compared to untreated controls, consistent with previous reports identifying oxidative stress as a key driver of melanogenesis [11]. Co-treatment with SJ196 at concentrations of 5 μg/mL and 10 μg/mL resulted in a dose-dependent reduction in ROS levels, demonstrating potent intracellular antioxidant activity. Oxidative stress is increasingly recognized as a central regulatory factor in melanocyte biology. Antioxidants such as polyphenols and flavonoids have been reported to suppress melanin synthesis primarily through radical-scavenging mechanisms [25]. In this study, SJ196 exhibited strong antioxidant activity in the ABTS assay, comparable to that of known radical scavengers such as quercetin and resveratrol [26]. The consistency between the cell-free antioxidant assays and intracellular ROS suppression confirmed by DCF-DA and MitoTracker Red dual staining supports the conclusion that SJ196 functions as a potent redox modulator in melanocytes.

At the signaling level, α-MSH stimulation in B16F10 cells rapidly increased the phosphorylation of ERK, JNK, and p38 MAPKs without altering total protein levels. This observation aligns with prior findings that ROS amplify MAPK signaling, thereby enhancing MITF activation and melanin synthesis [34]. Remarkably, SJ196 treatment significantly and dose-dependently attenuated the phosphorylation of all three MAPKs, indicating that its effects extend beyond direct antioxidant activity to the inhibition of redox-sensitive signaling cascades. Consistent with MAPK inhibition, SJ196 downregulated MITF expression at both the mRNA and protein levels, as shown by qPCR and Western blot analyses. Given that MITF is a master transcriptional regulator of melanogenic genes [44], its suppression explains the observed reductions in TYR, TRP-1, and TRP-2 expression in treated cells.

Both intracellular and extracellular melanin measurements demonstrated a dose-dependent reduction in pigment content in SJ196-treated cells compared to the α-MSH group, indicating effective suppression of melanin synthesis and secretion. These biochemical results were further corroborated using a three-dimensional human MelanoDerm™ skin model, which closely mimics human pigmentation. In this system, α-MSH reduced the L* value (indicating darkening), whereas co-treatment with SJ196 restored brightness in a concentration-dependent manner.

Taken together, this study demonstrates that 6-isoprenylindole-3-carboxylic acid (**1**), a prenylated indole alkaloid derivative obtained from the marine-derived *Streptomyces* sp. APA-053 exhibits significant antioxidant and anti-melanogenic activity. SJ196 exerts both antioxidant and signal-regulatory effects during melanogenesis, providing compelling evidence for its potential as a promising therapeutic agent for hyperpigmentation disorders and as a novel candidate ingredient for skin-whitening applications.

## Data Availability

The data supporting the reported results are contained within the manuscript and its Supporting Information.

## Supplementary Materials

The following supporting information can be downloaded at: https://www.mdpi.com/article/10.3390/md23120448/s1, Figure S1: ^1^H NMR Spectrum of 6-isoprenylindole-3-carboxylic acid (**1**) in methanol-*d*_4_; Figure S2: ^13^C NMR Spectrum of 6-isoprenylindole-3-carboxylic acid (**1**) in methanol-*d*_4_.
