# Supplementary material for: 6-Isoprenylindole-3-carboxylic Acid with an Anti-Melanogenic Activity from a Marine-Derived Streptomyces sp. APA-053
Source: Mar Drugs. 2025 Nov 21;23(12):448. doi: 10.3390/md23120448 (PMC12734698; doi:10.3390/md23120448)
Supplement: Supplementary file 1 [file marinedrugs-23-00448-s001.zip › marinedrugs-3973630-supplementary.pdf]

Supplementary Information

6-Isoprenylindole-3-carboxylic acid with an Anti-Melanogenic Activity from a Marine-Derived *Streptomyces* sp. APA-053

Minjeong Kim [1,†], Eun-Young Lee [1,†], Ga-Eun Shin [1], Jungwook Chin [2], Hongchan An [3,4], Sang-Jip Nam [5,6*] and Kyung-Min Lim [1,6,*]

[1] College of Pharmacy, Ewha Womans University, Seoul 03760, Republic of Korea; tyndall@ewha.ac.kr (M.K.); younglee0124@naver.com (E.-Y.L.); gaeun5136@ewhain.net (G.-E.S.)

[2] Cureverse, Inc., H2 Building, KIST, Seoul 02792, Republic of Korea; jwchin@cureverse.co.kr

[3] New Drug Development Center, Daegu-Gyeongbuk Medical Innovation Foundation, Daegu 41061, Republic of Korea

[4] College of Pharmacy and Institute of Pharmaceutical Sciences,, CHA University, 120 Haeryong-ro, Pocheon 11160, Republic of Korea; hongchanan@cha.ac.kr

[5] Department of Chemistry and Nanoscience, Ewha Womans University, Seoul 03760, Republic of Korea

[6] Graduate Program in Innovative Biomaterials Convergence, Ewha Womans University, Seoul 03760, Republic of Korea

* Correspondence: sjnam@ewha.ac.kr (S.-J.N.); kmlim@ewha.ac.kr (K.-M.L.); Tel.: +82-2-3277-6805 (S.-J.N.); +82-2-3277-3055 (K.-M.L)

† These authors equally contributed to this work.

Table of Contents

Figure S1. ^{1}H NMR Spectrum of 6-isoprenylindole-3-carboxylic acid (**1**) in methanol-d_4 ········ **S4**

Figure S2. ^{13}C NMR Spectrum of 6-isoprenylindole-3-carboxylic acid (**1**) in methanol-d_4 ········ **S5**

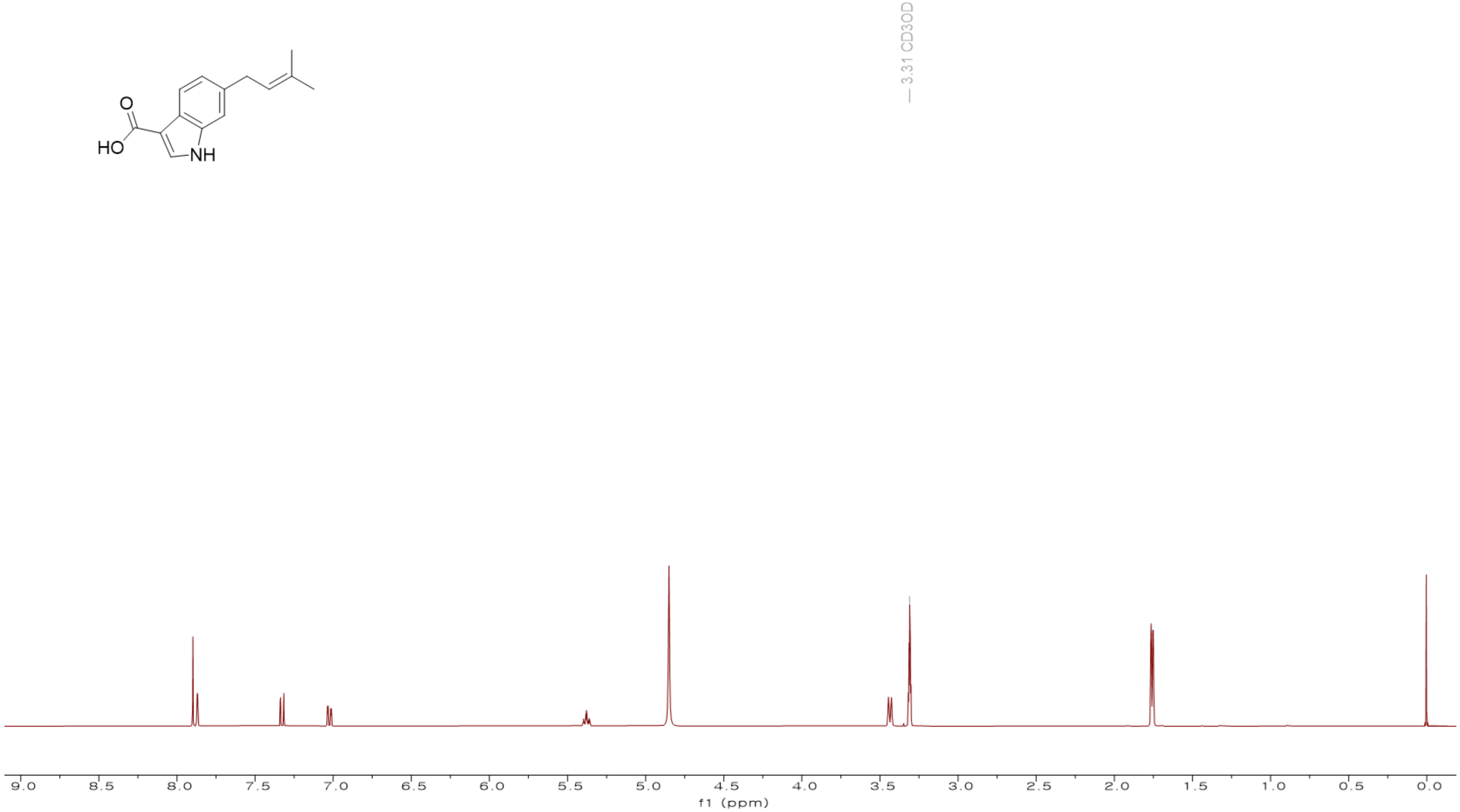

Figure S1. ^{1}H NMR spectrum of 6-isoprenylindole-3-carboxylic acid (**1**) in methanol-*d*4

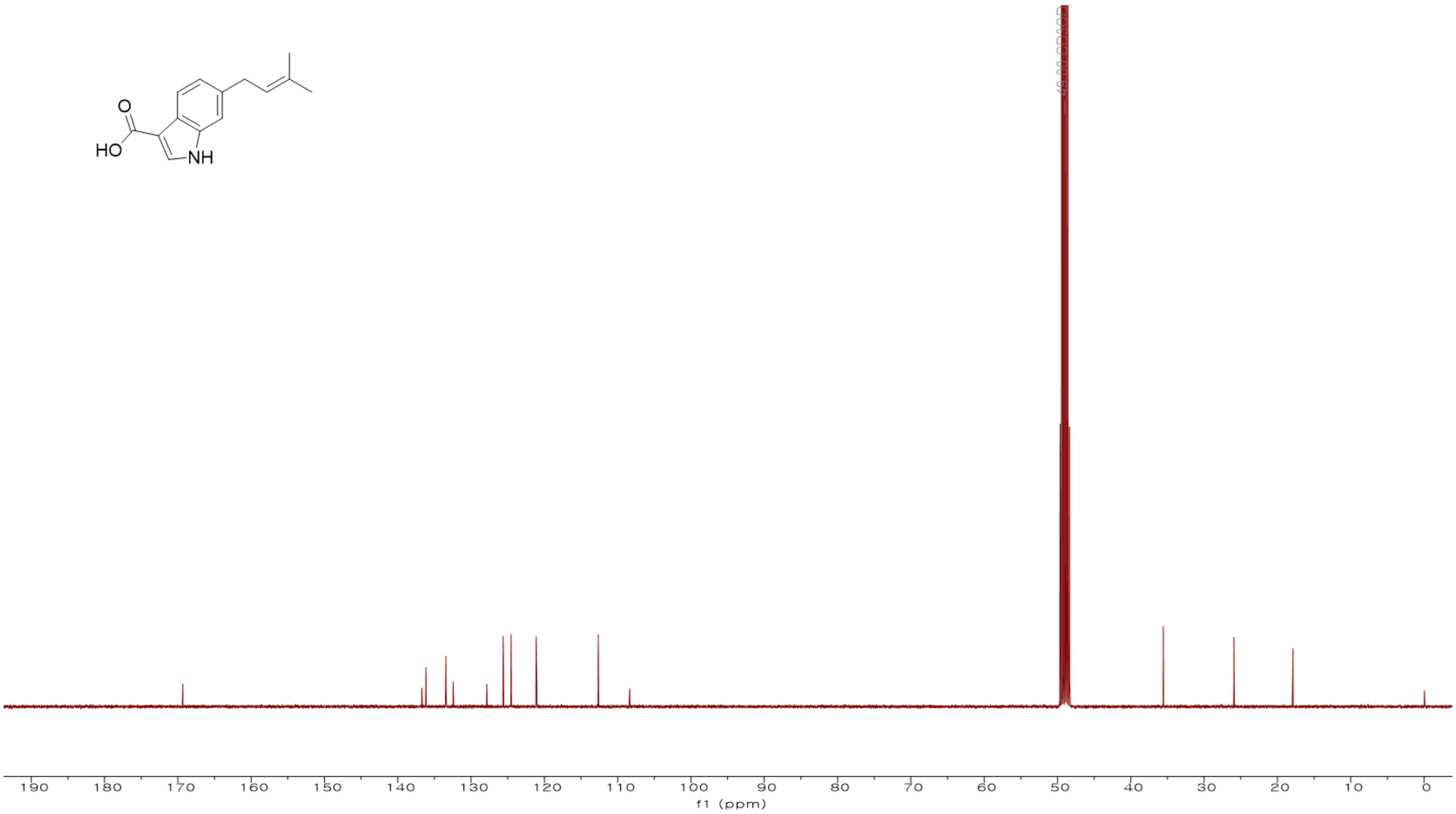

Figure S2. ^{13}C NMR spectrum of 6-isoprenylindole-3-carboxylic acid (**1**) in methanol-d_4
